# Surge of severe acute respiratory syndrome coronavirus 2 infections linked to single introduction of a virus strain in Myanmar, 2020

**DOI:** 10.1038/s41598-021-89361-7

**Published:** 2021-05-13

**Authors:** Myat Htut Nyunt, Hnin Ohnmar Soe, Kay Thi Aye, Wah Wah Aung, Yi Yi Kyaw, Aung Kyaw Kyaw, Theingi Win Myat, Aung Zaw Latt, Min Min Win, Aye Aye Win, Yin Min Htun, Khaing Mar Zaw, Phyu Win Ei, Kyaw Thu Hein, Lai Lai San, Nan Aye Thida Oo, Htin Lin, Nan Cho Nwe Mon, Khin Than Yee, Khin Lapyae Htun, Lynn Pa Pa Aye, Yamin Ko Ko, Thitsar Htet Htet Htoo, Kham Mo Aung, Hnin Azili, Soe Soe Han, Ni Ni Zaw, Su Mon Win, Wai Myat Thwe, Thin Thin Aye, Myat Su Hlaing, Wai Yan Minn, Pyae Phyo Thu, Hlaing Myat Thu, Zaw Than Htun

**Affiliations:** 1 Department of Medical Research, Ministry of Health and Sports, 5, Ziwaka Road, Dagon, Yangon, 11191 Republic of the Union of Myanmar; 2 Department of Microbiology, University of Medicine-2, Yangon, Republic of the Union of Myanmar

**Keywords:** Genetics, Microbiology, Molecular biology, Diseases, Molecular medicine

## Abstract

Severe acute respiratory syndrome coronavirus 2 (SARS-CoV-2) infection is a major health concern globally. Genomic epidemiology is an important tool to assess the pandemic of coronavirus disease 2019 (COVID-19). Several mutations have been reported by genome analysis of the SARS-CoV-2. In the present study, we investigated the mutational and phylogenetic analysis of 30 whole-genome sequences for the virus's genomic characteristics in the specimens collected in the early phase of the pandemic (March–June, 2020) and the sudden surge of local transmission (August–September, 2020). The four samples in the early phase of infection were B.6 lineage and located within a clade of the samples collected at the same time in Singapore and Malaysia, while five returnees by rescue flights showed the lineage B. 1.36.1 (three from India), B.1.1 (one from India) and B.1.80 (one from China). However, there was no evidence of local spread from these returnees. Further, all 19 whole-genome sequences collected in the sudden surge of local transmission showed lineage B.1.36. The surge of the second wave on SARS-CoV-2 infection was linked to the single-introduction of a variant (B.1.36) that may result from the strict restriction of international travel and containment efforts. These genomic data provides the useful information to disease control and prevention strategy.

## Introduction

The novel respiratory coronavirus disease (COVID-19), caused by severe acute respiratory syndrome coronavirus 2 (SARS-CoV-2), has been a source of major concern globally. In Myanmar, the first cases of COVID-19 were reported in March 2020, and only a few local cases were reported until the end of July 2020 as the result of the effective measures to contain the virus. These efforts contained a 21-day strict-quarantine policy from all returnees and contacts with confirmed cases; all contacts were checked by real-time PCR on the first week and before 21-days to exclude the infection, all confirmed cases were hospitalized and monitored until two consecutive negative by RT-PCR and stay-home order in high local cases detected. Moreover, since March 2020, restrictions on international flights have been started to prevent the introduction of the virus from other countries. Only Myanmar citizen-returnees by rescue flight were selectively allowed, and they were checked for two times swab examinations by molecular method within 21 days quarantine period after arrival. Consequently, only 379 confirmed cases were reported by August 18, 2020, in Myanmar^1^. However, after August 19, a sudden increase in local cases without a history of contact with known cases was reported in Rakhine State, western Myanmar. Within a week, local transmission was reported in the megacity, Yangon. Up to September 30, 2020, a total of 13,373 confirmed cases were recorded, with 310 deceased^2^.

The molecular epidemiology of the SARS-CoV-2 provides valuable information to formulate the strategy for prevention and control of the disease^3,4^. Mutational analysis and phylogenetic studies revealed that the virus originated from Wuhan was replaced by mutant variants in many countries^5^. However, up to now, no known study on genomic analysis of SARS-CoV-2 was documented in Myanmar.

Preliminary data with a history of traveling abroad, the imported confirmed cases from returnees indicated that different variants from various geographical territories had been introduced into Myanmar. We report the whole genome sequence analysis of SARS-CoV-2 detected in Myanmar to provide genetic evidence on the spread and dynamics of virus dissemination in this country.

## Methods

### Ethics statement

The study was reviewed and approved by the institutional review board (IRB) of the Department of Medical Research (Approval ID: Ethics/DMR/2020/073). In this study, we used the remaining anonymous samples for the diagnosis purpose; informed consent was waived by IRB. The study was registered at Myanmar Health Research Registry with ID: PLRID-00476_V5. All procedures were conducted according to the institutional guideline on responsible conduct of research.

### Study population

This study used the confirmed positive remaining nasopharyngeal swab samples after molecular diagnosis. According to the ARTIC protocol^6^, samples with low Ct value (< 30 cycles in all targeted genes by real-time PCR) were involved in this. Based on the daily confirmed positive COVID-19 rate, the early phase of the pandemic wave was defined as the duration before August-15, 2020, and that afterward, the sudden surge of infection was observed as the second wave in Myanmar. Sampling was done among the low Ct samples by randomly selecting returnees and locally reported cases. We selected five locally reported samples in the early phase of the pandemic wave and five samples from returnees from China and India. A total nine samples from the sudden surge of infection in Rakhine State and one returnee from the Philippines in August 2020, and another ten samples, in which nine of them were deceased cases in Yangon, were collected in September 2020 (Supplementary Table 1, Supplementary Fig. 1).

### RNA extraction and confirmation of the infection

We follow the procedures for whole-genome sequencing as described previously^7^. Briefly, nucleic acid was extracted from nasopharyngeal swab using QIAGEN QIAamp Viral RNA Mini Kit according to the manufacture's instruction. The SARS-CoV-2 infection in the samples was confirmed by the novel coronavirus (2019-nCoV) Nucleic Acid Diagnostic Kit (Sansure Biotech, Republic of China) using BioRad CFX96 Touch real-time PCR detection system according to the manufacturer's instruction.

### Sequencing procedures

We performed the amplification of the SARS-CoV2 whole-genome according to the ARTIC nCo-V-2019 protocol^6^. Briefly, cDNA was synthesized using the GoScript Reverse Transcriptase Kit (Promega). Amplification was done using Q5 hot-start high-fidelity DNA polymerase (NEB). One negative control was included in each amplification using the primer-pools. The amplicon was checked by capillary electrophoresis (LabChip GXTouch 24 nucleic acid analyzer). The negative control must be no visible band in electrophoresis in every amplification to exclude the contamination. Overlapping amplicons of 400 bp were purified by Illumina sample purification bead (Illumina, USA). The cleanup amplicons were quantified by Qubit3 dsDNA HS (High sensitivity) Assay Kit (Invitrogen) and normalized before library preparation by Nextera DNA Flex Library preparation Kit (Illumina). We used 500 ng input of amplified DNA for library preparation. The library has been checked quality by capillary electrophoresis and quantified by Qubit3. Normalization was carried out before pooling on ten samples in each run. These pooled samples were denatured by NaOH, and 11 pmol libraries were loaded with 10% PhiX spike control to run 151 paired-end in MiSeq next-generation sequencer.

### Sequence-analysis

The output sequences were proceeded for reads quality control by BBDuk and BBMap (BBTools 38.57). The sequences were checked by the genome detective virus tool (https://www.genomedetective.com/)^8^. Alignment against the SARS-CoV-2 reference genome sequence (NC_045512.2) was conducted. The variant calling was done by CoV-GLUE (http://cov-glue.cvr.gla.ac.uk/)^9^ and NextClade v0.5.0 (https://clades.nextstrain.org/)^10^ to assign the clade. The SARS-CoV-2 lineages were assessed by Pangolin COVID-19 Lineage Assigner (https://pangolin.cog-uk.io)^11^. The maximum likelihood phylogeny was done after aligned with reference sequence Wuhan-Hu-1 (NC_045512.2) and visualized by iTOL v5.6.3 (https://itol.embl.de/).

We submitted our sequences to NCBI SRA with accession numbers SAMN15733924-15733932, SAMN15722976, SAMN15921629-SAMN15921638 and SAMN16362251-SAMN16362260. The sequences were also deposited at GISAID (https://www.gisaid.org/).

## Result

We analyzed 30 nasopharyngeal swab specimens from patients with COVID-19 to conduct whole-genome analysis. Reads were aligned to the reference genome and consensus sequences were generated. After trimming the low-quality short reads and adapter (quality control), the median number of the reads was 2,228,704 with a mean sequencing depth of 8376-fold, covering more than 99% of the genome in all samples. We have identified average 12 mutations in which contains 30 synonymous, 34 non-synonymous substitutions, and five mutations at 5′UTR site along the whole genome (Table 1).

**Table 1:** Common mutations detected in 30 whole-genome sequences in Myanmar, 2020.

Nucleotide change (amino acid change)	Site of mutation	Local cases (March–July) n = 5	Local cases (August–September) n = 19	Cases from returnees n = 6
C241T	5′UTR	1/5	19/19	6/6
C3037T (synonymous mutation)	ORF1a	1/5	4/19	6/6
C14408T (P314L)	ORF1b	1/5	19/19	6/6
G18756T (synonymous mutation)	ORF1b	0/5	19/19	0/6
C18877T (synonymous mutation)	ORF1b	0/5	19/19	3/6
C22444T (synonymous mutation)	Spike	0/5	19/19	6/6
A23403G (D614G)	Spike	1/5	19/19	6/6
C23929T (synonymous mutation)	Spike	4/5	0/19	0/6
G25494T (synonymous mutation)	ORF3a	0/5	19/19	0/6
G25563T (Q57H)	ORF3a	0/5	19/19	3/6
C26735T (synonymous mutation)	M	0/5	19/19	3/6
C28311T (P13L)	N	4/5	0/19	0/6
G2881A, G2882A, G2883C (G204R)	ORF14	1/5	0/19	3/6
C28854T (S194L/Q41^a^)	ORF14	0/5	19/19	3/6

*ORF* open reading frame, *M* membrane gene, *N* nucleocapsid protein.

^a^Stop translation.

Among the five local cases in the early pandemic spread, four were B.6 lineage (PANGOLIN analysis: https://pangolin.cog-uk.io/), and one was B1.1 lineage. The three returnees from India were B.1.36.1, and one was B.1.1. Furthermore, we detected only one sample in local transmission cases in before the August 2020, with D614G mutation in spike protein altogether with Trinucleotide-Bloc Mutation, 28,881–28,883 GGG>AAC (R203K and G204R) at nucleoprotein (N) gene. The mutational analysis on the samples of returnees from India (four cases), China (one case), and the Philippines (one case) showed all were G variant (D614G mutant).

In our analysis, nine samples from Rakhine State and ten samples from Yangon City were included during the sudden surge of the cases. Whole-genome analysis on all these 19 samples showed B1.36 lineage. There were the same 11 mutations in all 19 samples (Table 1). All showed the synonymous mutation G18756T in ORF1b. Moreover, we observed C241T in 5′UTR, C3037T synonymous mutation at ORF1a, C14408T (P314L) at ORF1b, C18877T synonymous mutation at ORF1b, C22444T synonymous mutation at Spike, G25494T synonymous mutation at ORF3a, G25563T (Q57H) at ORF3a, C26735T in membrane protein, C28854T (S194L) at nucleoprotein in all samples taken from the sudden surge of local transmission (Table 1).

The phylogenetic analysis revealed that multiple introductions of the SARS-CoV-2 are possible in the early phase of the pandemic wave in Myanmar. At that time, locally reported cases were located at the same clade with Singapore and Malaysia, and returnee samples were identified at the same clade with India. However, a single introduction was observed in the pandemic wave's sudden surge that were situated at the same clade with the sequences of Bangladesh and India. Moreover, SARS-CoV-2 variants in deceased cases were not different from those in recovery cases.

**Figure 1:**
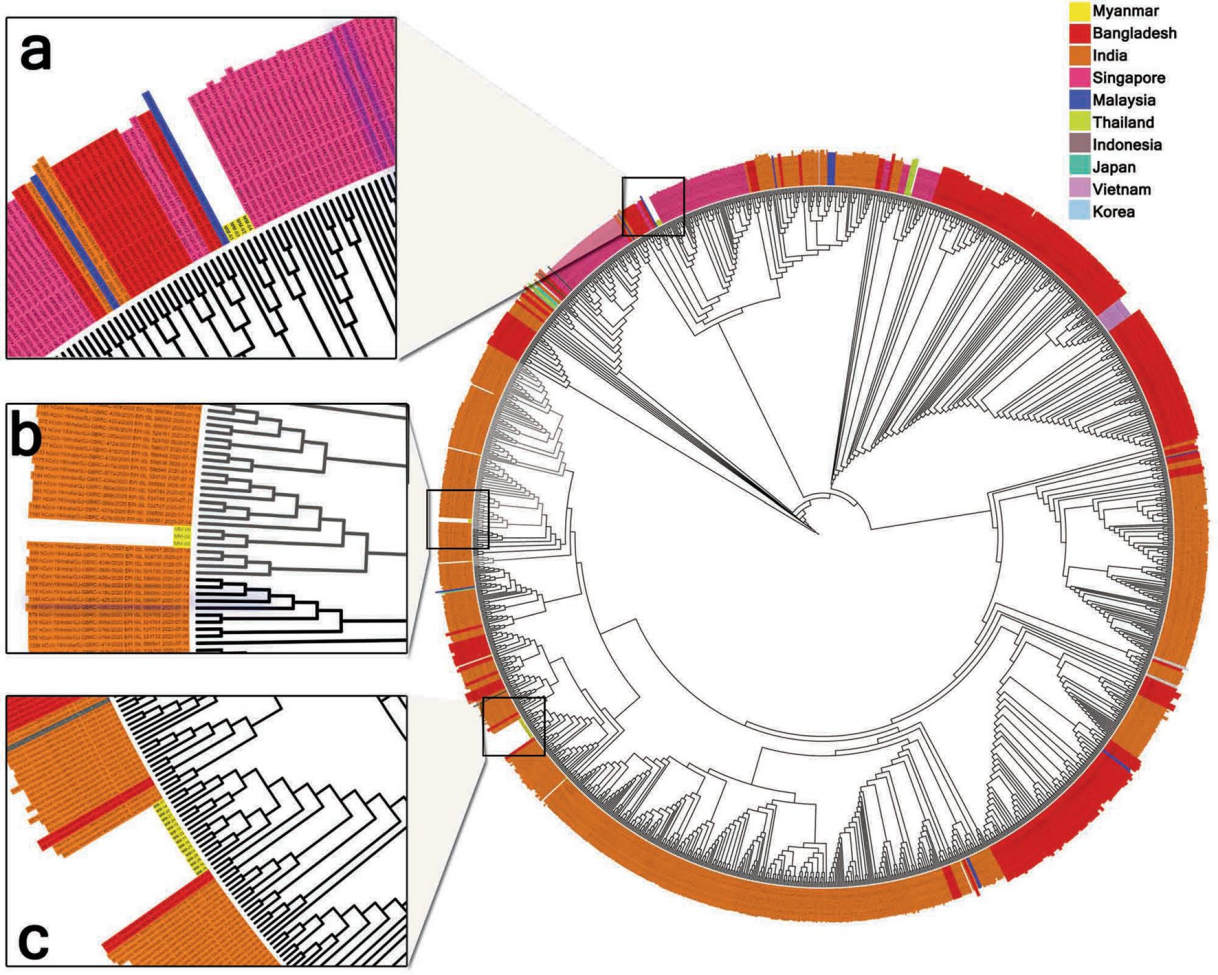
Maximum likelihood analysis clades of the 30 whole-genomes on SARS-CoV-2 in Myanmar, 2020 and sequences deposited at GISAID within July-1 to August-31, 2020 from India (820 sequences), Bangladesh (240 sequences), Thailand(5 sequences), Malaysia (13 sequences), Singapore(148 sequences), Indonesia (3 sequences), Japan (3 sequences), and Korea (3 sequences) with NC 045512.2 (Wuhan-Hu-1) as a reference. All sequences from local cases in Myanmar before the August-2020 were closely related to the Singapore clades (**a**). All sequences after August-2020 were appeared at the same clade with India and Bangladesh (**b**, **c**).

Phylogenetic analysis revealed that all four locally reported cases in the early part of the pandemic wave in Myanmar were observed at the same clade as the cases reported in Singapore and Malaysia, and one was identified at the same clade with India. However, all cases collected at the sudden surge of infection showed B.1.36 and were located at the same clade in India and Bangladesh (Fig. 1).

## Discussion

Genetic epidemiology provides useful information for the prevention and control of diseases. Whole-genome data can be applied for disease tracking in local transmission cases providing genomic evidence to policymakers. The genomic studies revealed that the SARS-CoV-2 mutation rate is similar to that of the other RNA viruses affecting the spread of the diseases globally^12^. Genomic surveillance is important to know the transmission of the disease by further action for containment, control, and prevention of the disease^13^.

Among the reported mutations, the non-synonymous mutation D614G was firstly observed in early 2020 but spread widely within a few weeks in many countries. The Spike D614G mutation is reputed to SARS-CoV-2 infection in vitro, suggesting it enhances viral infectivity, resulting in increased G variant globally^14,15^.

In the present study, only one GR variant (D614G in spike protein and G204R in ORF14) case was observed in local transmission cases in the early pandemic wave of infection. Among the returnees, GH variants (D614G and Q57H in ORF3a) from India and GR variants from China and the Philippines were observed. Because of the strict quarantine rules, infections in these returnees might not be spread locally in Myanmar.

Lineage B.6 was mainly detected in Southeast Asia in March and April 2020^16^. In our study, the local transmission cases had a history of primary and secondary contact with confirmed cases returned from Singapore. Among the samples taken from the returnees from India in April and May, we identified Lineage B.1.36.1. Meanwhile, the samples from China and Philippines' returnees showed Lineage B.1.80 (China) and B.1.1 (Philippine). According to the PANGOLIN analysis, B.1.36.1 is an Indian lineage circulating in India. The SARS-CoV-2 detected from the persons coming back from India showed B.1.36.1 lineage. These two returnees from India were kidney transplant receipt and donors, and they were hospitalized until two consecutive negative RT-PCR results. Therefore, there may be no further spread from these cases in Myanmar.

However, after August 15, a sudden increase in local transmission without any known contact history with confirmed cases was firstly reported in Rakhine State, western Myanmar, and later in Yangon megacity and other regions in Myanmar. Our analysis on 19 samples in this sudden surge of infection showed GH variant, B.1.36 lineage only with a similar mutation pattern.

Mutation at the 5′UTR region may affect the viral transcription and replication, leading to the viral infection cycle speed^17,18^. In our study, all D614G variants were coexistent with C241T at the 5′UTR. Although the role of that mutation, G18756T is still unclear, it was reported in India and the second time infection sample in South America^19^.

Among all mutations detected in the present study, the leader sequence mutation C241T is co-evolved with three important mutations, C3037T, C14408T, and A23403G, which result in amino acid mutations in nsp3 (synonymous mutation), RNA primase (P323L), and spike glycoprotein (S protein, D614G), respectively. These co-mutations are critical proteins for RNA replication and for binding to ACE2 receptor^20^. However, the virulence of the infection might not be related to the variants detected in this present study as the genetic characteristics of the SARS-CoV-2 of deceased cases were similar to that of recover cases.

Myanmar limits international travel, and only returnees of Myanmar citizens were allowed with limitations by relief flights. Consequently, only a few local cases were observed in the early pandemic wave before August 2020, although multiple introductions of the SARS-CoV-2 were observed. Despite the containment efforts, a single introduction of GH variant, probably from India or Bangladesh (Lineage B.1.36), was observed and spread, causing a sudden surge of COVID-19 wave in late August 2020 in Myanmar.

One limitation of our study is that only a subset of the laboratory-confirmed samples was included. A further study focusing on molecular surveillance to better understand the SARS-CoV-2 in Myanmar should be carried out.

## Conclusion

Mutational and phylogenetic analysis on whole-genome data in our study suggests multiple introductions of the SARS-CoV-2 in Myanmar. Since March 2020, local transmission of SARS-CoV-2 was reported. Still, genomic data indicated that early local transmission cases were B.1.6 lineage, which differed from the samples taken from the sudden surge of local transmission cases after August 2020 (B.1.36 lineage). Although the actual impact on these strains on a different aspect of pathogenicity and virulence is undetermined, dynamics of the viral sequences related to the sudden surge of the infection should be taken into deep concern in control of pandemic COVID-19.

## Supplementary Information

Supplementary Figure Legend.

Supplementary Figure 1.

Supplementary Table 1.
