# Supplementary material for: Surge of severe acute respiratory syndrome coronavirus 2 infections linked to single introduction of a virus strain in Myanmar, 2020
Source: Sci Rep. 2021 May 13;11:10203. doi: 10.1038/s41598-021-89361-7 (PMC8119731; doi:10.1038/s41598-021-89361-7)
Supplement: Supplementary file 1 — Supplementary Figure Legend. [file 41598_2021_89361_MOESM1_ESM.docx]

**Supplementary Figure Legend**

Supplementary Figure 1. Site of sample collection: Yangon Region and Rakhine State we shown with red dot. The map was created by Adobe Photoshop CS3, using the template map generated by QGIS v3.10.
