# Supplementary figures and images for: Surge of severe acute respiratory syndrome coronavirus 2 infections linked to single introduction of a virus strain in Myanmar, 2020
Source: Sci Rep. 2021 May 13;11:10203. doi: 10.1038/s41598-021-89361-7 (PMC8119731; doi:10.1038/s41598-021-89361-7)

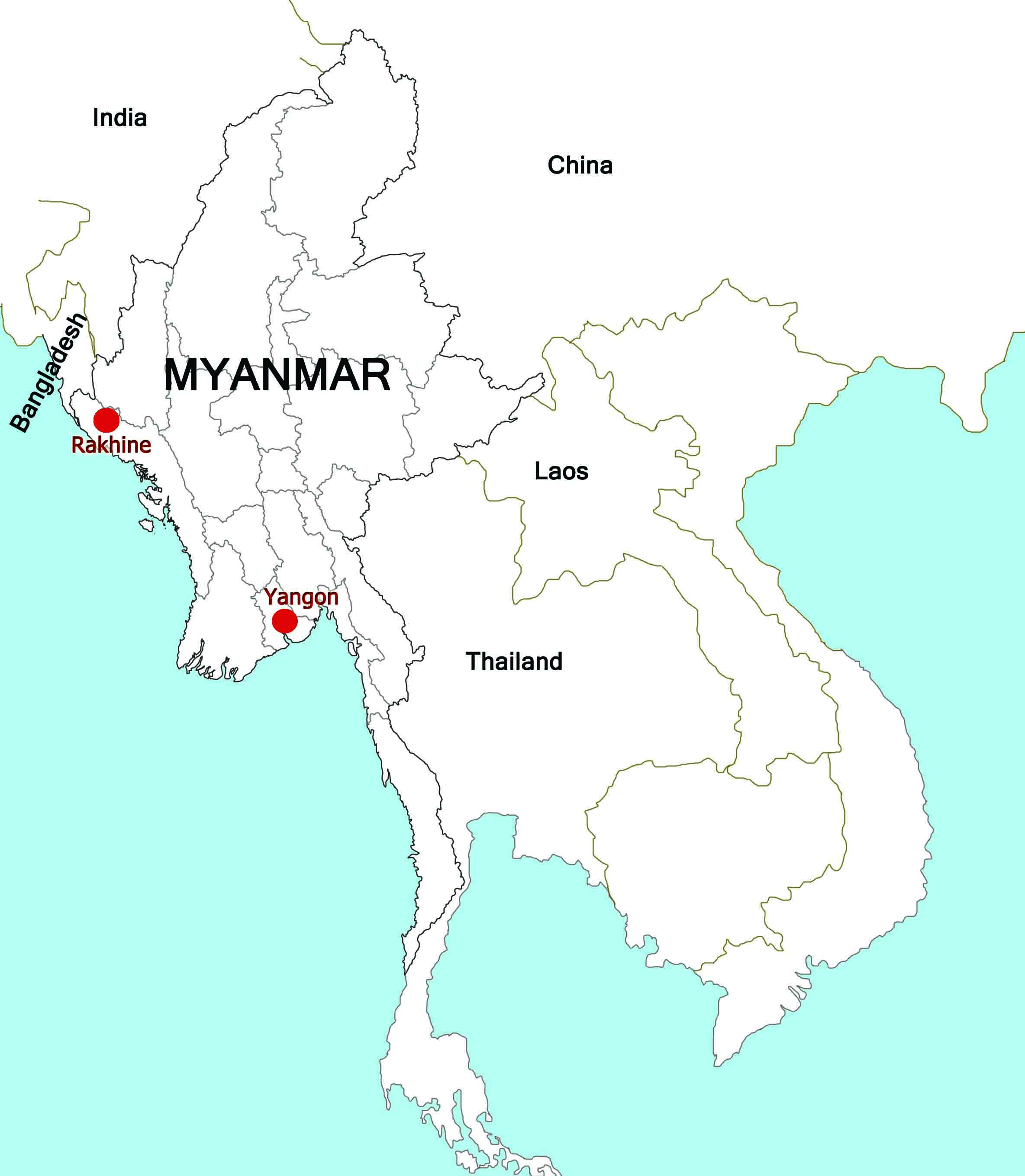

Supplement: Supplementary file 2 — Supplementary Figure 1. [file 41598_2021_89361_MOESM2_ESM.jpg]
