# Supplementary material for: Surge of severe acute respiratory syndrome coronavirus 2 infections linked to single introduction of a virus strain in Myanmar, 2020
Source: Sci Rep. 2021 May 13;11:10203. doi: 10.1038/s41598-021-89361-7 (PMC8119731; doi:10.1038/s41598-021-89361-7)
Supplement: Supplementary file 3 — Supplementary Table 1. [file 41598_2021_89361_MOESM3_ESM.docx]

**Surge of Severe Acute Respiratory Syndrome Coronavirus 2 Infections Linked to Single Introduction of a Virus Strain in Myanmar, 2020**

Myat Htut Nyunt^1^, Hnin Ohnmar Soe^1^, Kay Thi Aye^1^, Wah Wah Aung^1^, Yi Yi Kyaw^1^, Aung Kyaw Kyaw^1^, Theingi Win Myat^1^, Aung Zaw Latt^1^, Min Min Win^1^, Aye Aye Win^1^, Yin Min Htun^1^, Khaing Mar Zaw^1^, Phyu Win Ei^1^, Kyaw Thu Hein^1^, Lai Lai San^1^, Nan Aye Thida Oo^1^, Htin Lin^1^, Nan Cho Nwe Mon^1^, Khin Than Yee^1^, Khin Lapyae Htun^1^, Lynn Pa Pa Aye^1^, Yamin Ko Ko^1^, Thitsar Htet Htet Htoo^1^, Kham Mo Aung^1^, Hnin Azili^1^, Soe Soe Han^1^, Ni Ni Zaw^1^, Su Mon Win^1^, Wai Myat Thwe^1^, Thin Thin Aye^1^, Myat Su Hlaing^2^, Wai Yan Minn^2^, Pyae Phyo Thu^2^, Hlaing Myat Thu^1^ and Zaw Than Htun^1^

**Affiliations:**

1 Department of Medical Research, Ministry of Health and Sports, Yangon, Republic of the Union of Myanmar

2 Department of Microbiology, University of Medicine-2, Yangon, Republic of the Union of Myanmar

**Supplementary Table 1.** Demographic characteristics of the sequenced patients with severe acute respiratory syndrome coronavirus 2, Myanmar, 2020

| ID | Age | Sex | Place | Contact history with known positive cases | Foreign country travel history | Diagnosis date (2020) | Outcome | Nextstrain clade | GISAID clade | PANGOLIN Lineage |
| --- | --- | --- | --- | --- | --- | --- | --- | --- | --- | --- |
| MM1 | 35 | M | Yangon | Yes | No | 24-Apr | Recover | 19A | L | B.6 |
| MM2 | 31 | F | Yangon | Yes | No | 24-Apr | Recover | 19A | L | B.6 |
| MM3 | 53 | M | Yangon | Yes | No | 24-Apr | Recover | 19A | L | B.6 |
| MM4 | 48 | M | Yangon | No | No | 3-May | Recover | 19A | L | B.6 |
| MM5 | 28 | F | Yangon | Yes | No | 7-May | Recover | 20B | GR | B.1.1 |
| MM6 | 41 | F | Yangon | No | Yes (India) | 25-May | Recover | 20A | GH | B.1.36.1 |
| MM7 | 29 | F | Yangon | No | Yes (China) | 2-Jun | Recover | 20A | GH | B.1.80 |
| MM8 | 63 | F | Yangon | No | Yes (India) | 26-May | Recover | 20A | GH | B.1.36.1 |
| MM9 | 66 | M | Yangon | No | Yes (India) | 26-May | Recover | 20A | GH | B.1.36.1 |
| MM10 | 40 | F | Yangon | No | Yes (India) | 30-May | Recover | 20B | GR | B.1.1 |
| MM11 | 22 | M | Sittwe | Yes | No | 21-Aug | Recover | 20A | GH | B.1.36 |
| MM12 | 58 | M | Sittwe | Yes | No | 21-Aug | Recover | 20A | GH | B.1.36 |
| MM13 | 28 | M | Sittwe | Yes | No | 21-Aug | Recover | 20A | GH | B.1.36 |
| MM14 | 38 | F | Sittwe | No | No | 20-Aug | Recover | 20A | GH | B.1.36 |
| MM15 | 29 | F | Sittwe | No | No | 20-Aug | Recover | 20A | GH | B.1.36 |
| MM16 | 83 | M | Sittwe | No | No | 20-Aug | Recover | 20A | GH | B.1.36 |
| MM17 | 28 | F | Sittwe | Yes | No | 20-Aug | Recover | 20A | GH | B.1.36 |
| MM18 | 43 | M | Sittwe | Yes | No | 21-Aug | Recover | 20A | GH | B.1.36 |
| MM19 | 50 | F | Sittwe | Yes | No | 19-Aug | Recover | 20A | GH | B.1.36 |
| MM20 | 21 | F | Yangon | No | Yes(Philippine) | 19-Aug | Recover | 20B | GR | B.1.1 |
| MM21 | 75 | M | Yangon | No | No | 12-Sep | Deceased | 20A | GH | B.1.36 |
| MM22 | 84 | F | Yangon | No | No | 13-Sep | Deceased | 20A | GH | B.1.36 |
| MM23 | 58 | M | Yangon | Yes | No | 16-Sep | Deceased | 20A | GH | B.1.36 |
| MM24 | 67 | M | Yangon | Yes | No | 16-Sep | Deceased | 20A | GH | B.1.36 |
| MM25 | 24 | M | Yangon | No | No | 14-Sep | Deceased | 20A | GH | B.1.36 |
| MM26 | 74 | M | Yangon | No | No | 17-Sep | Deceased | 20A | GH | B.1.36 |
| MM27 | 75 | F | Yangon | Yes | No | 17-Sep | Deceased | 20A | GH | B.1.36 |
| MM28 | 48 | F | Yangon | No | No | 17-Sep | Deceased | 20A | GH | B.1.36 |
| MM29 | 23 | F | Yangon | No | No | 17-Sep | Deceased | 20A | GH | B.1.36 |
| MM30 | 53 | M | Yangon | No | No | 28-Sep | Recover | 20A | GH | B.1.36 |
